# Goal Concordant Care: A Cross-Sectional Study Evaluating Correlates of Concordant Care and Association With Satisfaction and Outcomes

**DOI:** 10.1177/15589447241279458

**Published:** 2024-09-26

**Authors:** Kaitlyn R. Julian, Jeffrey W. Kwong, Chelsea Leversedge, Thompson Zhuang, Nicole Schroeder, Robin N. Kamal, Lauren M. Shapiro

**Affiliations:** 1 University of California, San Francisco, USA; 2 Stanford University, Redwood City, CA, USA; 3 University of Pennsylvania, Philadelphia, USA

**Keywords:** equity, socioeconomic status, hand, anatomy, fracture/dislocation, diagnosis, wrist, health disparity

## Abstract

**Background:**

The concordance between patient and physician goals has been associated with improved outcomes in many chronic diseases. The purpose of this study was to evaluate the association between goal concordant care, patient satisfaction, and patient experience and to analyze factors associated with goal concordant care in hand and upper extremity surgery.

**Methods:**

New patients who were 18 years or older were invited to participate. Goal concordant care was defined as the patient’s previsit treatment goal matching the primary treatment received. The χ^2^ tests were used to evaluate the association between goal concordant care and patient satisfaction and patient experience. We conducted univariable logistic regression to evaluate variables for their association with concordance and multivariable logistic regression for variables that were significantly associated in the initial analyses to evaluate their aggregate influence on concordance.

**Results:**

In total, 169 patients enrolled. The rate of goal concordant care was 62%; concordance was not associated with patient satisfaction or experience. Age, sex, English proficiency, health literacy, education level, employment and relationship status, pain self-efficacy, symptom duration, functional disability, and patient-centered decision-making were not associated with concordant care. Patients with annual income less than $50,000 had significantly higher odds of goal discordant care.

**Conclusion:**

Patients with lower income had more than 3 times the odds of receiving discordant care. However, discordant care was not associated with patient satisfaction or experience. Further studies on other pertinent outcomes are needed in orthopedic surgery (eg, treatment adherence). Known care disparities based on socioeconomic status may be mediated through care discordance and should be investigated.

## Introduction

High-quality health care includes care that incorporates and is responsive to individual patient values and preferences.^1^ As such, the National Academy of Medicine has identified goal concordant care, where the goals of the patient match the treatment, as a key priority,^2^ and goal concordant care has been proposed as a quality measure.^3^ Goal concordant care has been associated with positive impacts on health in patients with chronic conditions.^4^ For example, in an evaluation of adult patients with rheumatoid arthritis, goal concordance was associated with a higher rate of medication adherence. In hospitalized adults, goal concordant care was demonstrated to improve clinical outcomes, reduce patient and surrogate distress, and minimize health care costs.^2,5^ Goal concordance in hand surgery, however, has not been explored.

Measures of patient satisfaction and patient experience are increasingly being used to evaluate quality of care.^6,7^ Many patient-, surgeon-, and system-related factors have been demonstrated to influence patient satisfaction and patient experience ratings. For example, perceived physician empathy,^8^ older age,^9^ and time spent with the provider^10^ have been associated with increased patient satisfaction and patient experience scores, as well as improved patient outcomes in orthopedic surgery. On the contrary, psychosocial factors,^8^ longer wait times,^9^ and greater social deprivation^11^ appear to diminish these measures.

The association between goal concordance and patient satisfaction and experience has not been previously evaluated in hand and upper extremity surgery. Furthermore, it is unknown what patient factors are associated with goal concordance, which may be an important source of disparities in care delivery. As such, we asked, in the context of hand and upper extremity surgery, (1) Is there an association between goal concordant care and patient satisfaction and patient experience? (2) What factors are associated with goal concordant care?

## Materials and Methods

### Study Participants

This study was reviewed and approved for human subjects research as determined by our hospitals’ Institutional Review Boards. In this cross-sectional study, new patients who were 18 years or older presenting to the clinic of 3 fellowship-trained orthopedic hand surgeons at 2 academic hospital systems were invited to participate. If the patients spoke a language other than English, interpreters were used.

### Study Procedures

Before seeing the surgeon, patients created a rank order list of their top 3 treatment goals from a list of 6 options. The surgeons did not view the patients’ identified treatment goals prior to the clinic visit. The options consisted of scheduling surgery, receiving medication, receiving an injection, receiving information, receiving a brace/splint, and receiving imaging. This list was developed based on the most common treatment options offered by the 3 hand surgeons and was piloted with 10 patients to ensure comprehensiveness. After seeing the surgeon, patients ranked the primary, secondary, and tertiary treatments received from the same 6 options. All study data were managed using Research Electronic Data Capture database (REDCap, Vanderbilt University, Nashville, Tennessee). Goal concordant care was defined as the patient’s primary goal matching the primary treatment. We performed a sensitivity analysis with concordance defined as the patient’s primary goal being among the primary or secondary treatment received. Participants were dichotomized into concordant and discordant groups. We descriptively report the rates of concordant treatment.

### Measures

Patients completed additional surveys consisting of demographic information (age, sex, income, race/ethnicity, employment status, education, relationship status, insurance type), health literacy (Single-Item Literacy Screener [SILS]), English proficiency (US Census English proficiency question [Census-LEP]), symptom length, pain self-efficacy (pain self-efficacy questionnaire [PSEQ-2]), and functional disability (PROMIS Item Bank v2.0—Physical Function—Short Form 7a). During the visit, researchers (KRJ and CL) observed and scored each surgeon’s patient-centered decision-making ability as measured by the Observer OPTION5 tool.^12^ The 2 researchers completed Observer OPTION5 training and had an interrater reliability of 0.82 by observing 10 trial patient visits prior to the start of the study. Interrater reliability values between 0.75 and 0.9 indicate good reliability.^13^ After the visit, patients completed measures of patient satisfaction (Press Ganey Outpatient Medical Practice Survey [PGOMPS]) and patient experience (likelihood to recommend [LTR] scale).

### Health Literacy

The SILS was used to assess the health literacy of participants. The SILS was considered positive if scores were greater than 2, which indicate difficulty reading printed health-related material.^14^

### English Proficiency

The US Census English proficiency question (Census-LEP) was used to assess English proficiency. A threshold of less than “very well” was used, and we additionally asked patients for their language preferences at home and with medical care.^15^

### Functional Disability

The PROMIS Item Bank v2.0—Physical Function—Short Form 7a, a validated patient-reported outcome measure, was used to determine the functional disability of the patient. Lower PROMIS scores indicated a higher level of disability and lower functional status.^16^

### Pain Self-efficacy

The PSEQ-2 includes 2 items scored on a 7-point Likert scale which are added to form a total score ranging from 0 to 12.^17,18^ Higher scores indicate greater self-efficacy, which is defined as patients’ confidence in their ability to engage in activities of daily living despite their pain. A threshold of 10 was used, and scores less than 10 were indicative of less pain self-efficacy.^17,18^

### Patient-Centered Decision-Making Ability

The observer OPTION 5 tool is a validated instrument used to quantify a provider’s patient-centered decision-making ability.^12^ The tool consists of 5 items each scored on a scale from 0 to 4. A higher score indicates a higher level of patient involvement in the clinical decision-making process.

### Patient Satisfaction

Patient satisfaction was determined using the PGOMPS, which consists of 10 questions and a Likert scale of 5 response options: very poor, poor, fair, good, and very good. Patients were classified as dissatisfied with the encounter if their mean total score was less than the 33rd percentile, and patients were classified as satisfied if the mean total score was above the 33rd percentile. This cutoff was determined based on previously published methodology for Press Ganey satisfaction, which accounts for the potential ceiling effect of this scoring method.^9,19^ The 33rd percentile in our study was 0.98.

### Patient Experience

We used an LTR scale in which 1 was “extremely unlikely” and 5 “extremely likely.” Positive patient experience was classified as a score of 5 (“extremely likely to recommend”).^20^

### Data Analysis

The χ^2^ tests were used to evaluate the association between goal concordant care and patient satisfaction and patient experience. Logistic regression was employed to assess the association between concordance and each predictor variable. An initial univariate screen was employed with a threshold of *P* < .05, and variables identified in the screen were included in a multivariable regression model. Odds ratios are presented as point estimates with 95% confidence intervals. We additionally conducted a post hoc analysis investigating how patients’ previsit treatment goals and postvisit treatments varied by income status. Fisher exact test was used to evaluate the distribution of treatment preferences. An a priori power analysis demonstrated that to detect a 20% difference in patient experience, based on the LTR, 162 patients would be needed to achieve a power of 80% at an alpha of .05. All statistical analyses were performed using SAS OnDemand for Academics (Cary, North Carolina) and significance was set at *P* < .05.

## Results

We included 169 patients in our study (mean age 53; 48% men), and concordant care was achieved 62% of the time (Table 1). Leading goals included receiving information (60%), scheduling surgery (12%), and receiving imaging (12%). Leading treatments included receiving information (52%), imaging (18%), or an injection (11%).

**Table 1.** Patient Characteristics by Concordance, Discordance, and in Total.

Correlates of concordant care	Patient characteristics	Total N = 169	Concordant N = 105	Discordant N = 64	*P* value
Gender, No. (%)	Male Female	81 (47.9) 88 (52.1)	50 (47.6) 55 (52.4)	31 (48.4) 33 (51.6)	.918
Age, y, mean (SD)		52.9 (17.8)	52.6 (18.2)	53.3 (17.3)	.796
Limited English proficiency, No. (%)		26 (15.4)	13 (12.4)	13 (20.3)	.169
Limited health literacy, No. (%)		24 (14.2)	13 (12.4)	11 (17.2)	.387
Language spoken at home, No. (%)	English Other	144 (85.2) 25 (14.8)	94 (89.5) 11 (10.5)	50 (78.1) 14 (21.9)	.047*
Preferred language of medical care, No. (%)	English Other	157 (92.9) 12 (7.1)	101 (96.2) 4 (3.8)	56 (87.5) 8 (12.5)	.043*
Education^a^, No. (%)	High school	21 (12.4)	12 (11.4)	9 (14.1)	.504
Employment status, No. (%)	Full-time employed	81 (47.9)	49 (46.7)	32 (50.0)	.960
Income^a^, No. (%)	Less than $50,000 Greater than $50,000	38 (22.9) 128 (77.1)	15 (14.7) 87 (85.3)	23 (35.9) 41 (64.1)	.002*
Relationship status, No. (%)	Married	103 (61.0)	66 (62.9)	37 (57.8)	.426
Insurance type, No. (%)	Private Other	123 (66.5) 46 (33.5)	81 (77.1) 24 (22.9)	42 (65.6) 22 (34.4)	.105
Race/ethnicity, No. (%)	White/Caucasian	98 (58.0)	64 (61.0)	34 (53.1)	.318
Pain self-efficacy, No. (%)	Low High	89 (52.7) 80 (47.3)	54 (51.4) 51 (48.6)	35 (54.7) 29 (45.3)	.681
Patient satisfaction, No. (%)	Dissatisfied Satisfied	60 (35.5) 109 (64.5)	38 (36.2) 67 (63.8)	22 (34.4) 42 (65.6)	.811
Patient experience, No. (%)	Likely to recommend	145 (85.8)	90 (85.7)	55 (85.9)	.968
Symptom duration, mo, mean (SD)		20.8 (49.1)	18.1 (44.9)	25.3 (55.3)	.361
PROMIS PF score, mean (SD)		40.4 (11.7)	39.6 (11.7)	41.7 (11.5)	.267
OPTION5 score, mean (SD)		12.4 (3.62)	12.5 (3.54)	12.3 (3.77)	.795
Treating surgeon	Surgeon A Surgeon B Surgeon C	69 (40.8%) 45 (26.6%) 55 (32.5%)	41 (39.1%) 31 (29.5%) 33 (31.4%)	28 (43.8%) 14 (21.9%) 22 (34.4%)	.552

*Note. P* values are from univariate logistic regression. PROMIS PF = Patient-Reported Outcomes Measurement Information System Physical Function; OPTION 5 = Observing Patient Involvement in Shared Decision Making tool.

a N is discrepant due to overlapping data or incomplete responses.

* *P* < .05.

### Association of Goal Concordant Care With Patient Satisfaction and Patient Experience

Goal concordant care was not associated with patient satisfaction (PGOMPS, *P* = .99) or patient experience (LTR, *P* = .81). In addition, scheduling surgery or receiving an injection was not found to be associated with PROMIS scores (*P* = .11), patient satisfaction (*P* = .49), patient experience (*P* = 0.60), or OPTION5 scores (*P* = .66).

### Correlates of Goal Concordant Care

On primary and sensitivity analyses, age, sex, English proficiency, health literacy, education level, employment status, relationship status, pain self-efficacy, symptom duration, functional disability, and patient-centered decision-making were not associated with goal concordant care (Tables 1 and 2). Lower annual household income (less than $50,000) was associated with goal discordant care on primary analysis (*P* = 0.002) and remained significant in the sensitivity analysis (*P* = .001). Insurance type was found to be significant on sensitivity analysis (*P* = .042). In univariate logistic regression, patients with an annual household income less than $50,000 had an odds ratio of 3.25 for receiving discordant care (*P* = .002, 95% confidence interval, CI = 1.54-6.88). In addition, patients who spoke no English at home had an odds ratio of 2.39 for receiving discordant care (*P* = .047, 95% CI = 1.01-5.66), and patients who preferred their medical care in a language other than English had an odds ratio of 3.61 for receiving discordant care (*P* = .043, 95% CI = 1.04-12.5). On univariate logistic regression sensitivity analysis, patients with an annual income less than $50,000 had an odds ratio of 3.71 for receiving discordant care (*P* = .001, 95% CI = 1.67-8.21) and patients with other insurance than private had an odds ratio of 2.21 for receiving discordant care (*P* = .042, 95% CI = 1.03-4.80).

**Table 2.** Sensitivity Analysis of Patient Characteristics by Concordance, Discordance, and in Total.

Correlates of concordant care	Patient characteristics	Total N = 169	Concordant N = 132	Discordant N = 37	*P* value
Gender, No. (%)	Male Female	81 (47.9) 88 (52.1)	63 (47.7) 69 (52.3)	18 (48.6) 19 (51.4)	.921
Age, y, mean (SD)		52.9 (17.8)	52.1 (17.8)	53.1 (18.2)	.756
Limited English proficiency, No. (%)		26 (15.4)	20 (15.2)	6 (16.2)	.874
Limited health literacy, No. (%)		24 (14.2)	18 (13.4)	6 (16.2)	.692
Language spoken at home, No. (%)	English Other	144 (85.2) 25 (14.8)	132 (85.6) 19 (14.4)	31 (83.8) 6 (16.2)	.783
Preferred language of medical care, No. (%)	English Other	157 (92.9) 12 (7.1)	124 (93.9) 8 (6.1)	33 (89.2) 4 (10.8)	.327
Education^a^, No. (%)	High school	21 (12.4)	15 (11.4)	6 (16.2)	.706
Employment status, No. (%)	Retired	81 (47.9)	63 (47.7)	18 (48.7)	.970
Income^a^, No. (%)	Less than $50,000 Greater than $50,000	38 (22.9) 128 (77.1)	22 (17.1) 107 (82.9)	16 (43.2) 21 (56.8)	.001*
Relationship status, No. (%)	Married Other	103 (61.0)	84 (63.6) 48 (36.4)	19 (51.4) 18 (48.6)	.600
Insurance type, No. (%)	Private Other	123 (66.5) 46 (33.5)	101 (76.5) 31 (23.5)	22 (59.5) 15 (40.5)	.042*
Race/ethnicity^a^, No. (%)	White/Caucasian Other	98 (58.0)	78 (59.1) 54 (40.9)	20 (54.1) 17 (45.9)	.584
Pain self-efficacy, No. (%)	Low High	89 (52.7) 80 (47.3)	69 (52.3) 63 (47.7)	20 (54.1) 17 (45.9)	.848
Patient satisfaction, No. (%)	Dissatisfied Satisfied	60 (35.5) 109 (64.5)	46 (34.9) 86 (65.2)	14 (37.8) 23 (62.3)	.737
Patient experience, No. (%)	Likely to recommend	145 (85.8)	114 (86.4)	31 (83.8)	.692
Symptom duration, mo, mean (SD)		20.8 (49.1)	19.7 (51.2)	24.6 (40.8)	.595
PROMIS PF score, mean (SD)		40.4 (11.7)	39.6 (11.6)	43.2 (11.5)	.094
OPTION5 score, mean (SD)		12.4 (3.62)	12.5 (3.45)	12 (4.18)	.422
Treating surgeon	Surgeon A Surgeon B Surgeon C	69 (40.8%) 45 (26.6%) 55 (32.5%)	53 (40.2%) 37 (28.0%) 42 (31.8%)	16 (43.2%) 8 (21.6%) 13 (35.1%)	.738

*Note.* Concordance for the sensitivity analysis was defined as the patient’s primary goal being among the primary or secondary treatment received. PROMIS PF = Patient-Reported Outcomes Measurement Information System Physical Function; OPTION 5 = Observing Patient Involvement in Shared Decision Making tool.

a N is discrepant due to overlapping data or incomplete responses.

* *P* < .05.

On multivariate analysis, income remained a significant predictor of concordance, with an odds ratio of 2.76 for receiving discordant care (*P* = .012, 95% CI = 1.25-6.12). Language spoken at home (*P* = .55) and preferred language of medical care (*P* = .58) did not remain a significant predictor of concordance on multivariate analysis (Table 3). In addition, on sensitivity analysis, income remained a significant predictor of concordance (*P* = .015) and insurance type did not remain a significant predictor of concordance (*P* = .59) (Table 3).

**Table 3A.** Multivariate Regression Primary Analysis.

Correlates of Concordant Care	OR (95% CI)	*P* value
Income Less Than $50,000	2.76 (1.25, 6.12)	0.012*
Language Spoken at Home	1.40 (0.46, 4.23)	0.550
Preferred Language of Medical Care	1.56 (0.32, 7.64)	0.583

*Note.* OR = odds ratio; CI = confidence interval.

* *P* < .05.

We conducted a post hoc analysis investigating how patients’ previsit treatment goals and postvisit treatments varied by income status. Patients with an annual household income less than $50,000 were more likely to desire surgery, medication, imaging, or bracing (Table 4). By contrast, patients with a household income more than $50,000 desired information most often. After the visit, most higher income patients received information, as they had wanted, whereas those with lower income received a more even spread of treatment options (Table 4).

**Table 3B.** Multivariate Regression Sensitivity Analysis.

Correlates of Concordant Care	OR (95% CI)	*P* value
Income Less Than $50,000	3.22 (1.26, 8.26)	0.015*
Insurance Type	1.30 (0.51, 3.32)	0.589

*Note.* OR = odds ratio; CI = confidence interval.

* *P* < .05.

**Table 4.** Post Hoc Analysis of Patient’s Previsit and Postvisit Goals by Income Status.

Income status	Surgery	Injection	Medication	Information	Brace/splint	Imaging	Total
<50,000, No. (%)							38
Previsit Postvisit	6 (15.8) 4 (10.5)	2 (5.3) 3 (7.9)	3 (7.9) 1 (2.6)	14 (36.8) 13 (34.2)	5 (13.2) 4 (10.5)	8 (21.0) 13 (34.2)	
>50,000, No. (%)							131
Previsit Postvisit	14 (10.7) 12 (9.2)	9 (6.9) 16 (12.2)	2 (1.5) 3 (2.3)	88 (67.2) 74 (56.5)	5 (3.8) 9 (6.9)	13 (9.9) 17 (13.0)	

## Discussion

Care that is congruent with an individual’s goals and values is important for the delivery of high-quality and patient-centered care. The benefits of goal concordant care have been demonstrated in patients with rheumatologic conditions and in palliative care^4,21^; however, to date, no investigations have been conducted in orthopedic surgery. We demonstrate that lower income patients were more likely to receive discordant care. While concordance was not associated with patient satisfaction or experience, the association of income with goal discordant care illustrates a potential disparity and highlights opportunities for interventions to improve the delivery of equitable care in these populations.

Among patients presenting to orthopedic hand and upper extremity surgery clinic, the rate of goal concordant care in this investigation was 62%. This finding is similar to that of other studies evaluating different patient populations, including those examining goal concordant care in the outpatient setting that demonstrated goal concordance rates of 60% among patients with serious illness and 80% among patients with rheumatoid arthritis.^22^ Notably, we did not find goal concordant care to be associated with patient satisfaction or patient experience. Rane et al,^9^ in evaluating patient satisfaction in a hand surgery clinic, demonstrated that patients receiving an intervention (eg, scheduling surgery, receiving an injection) reported greater satisfaction. Although the driver of this association is unclear, it is possible this observation is due to the immediate effect of an injection, the perception they were taken seriously, or the patients’ concerns being affirmed. We analyzed the impact of receiving an intervention (eg, scheduling surgery, receiving an injection) and demonstrated that neither was associated with increased patient satisfaction or patient experience. Longitudinal investigation with other variables of patient experience is warranted to determine whether goal concordant care within orthopedic and/or hand surgery is associated with long-term patient satisfaction, patient experience, and/or optimal outcomes (eg, complication rates, return to work time). Future studies could employ qualitative analyses to more directly explore patient perceptions about treatment goals and concordant care.

Although goal concordance was not associated with patient satisfaction or experience, exploring factors that leads to goal discordance may have implications for the provision of equitable care. While patient income is not modifiable, it is important to recognize this as a potential contributor to discordant care. In the post hoc analysis, we found that lower income patients were more likely to request surgery, medication, bracing/splinting, and imaging compared with higher income patients who most often desired information. As such, the observed disparities may be due to differences in desired treatments that may exist before patients present to clinic. Evidence demonstrates that lower income patients access care further on in their disease processes, present more severely, more frequently consider the cost of treatment and the impact of time off work, and lack education surrounding their condition.^23-26^ Moreover, it is possible that the tendency for higher income patients to desire information reflects a greater ability to seek second opinions or the ability to weigh treatment options before committing to a particular treatment. This is consistent with other investigations that show patients of higher socioeconomic status are more likely to use second opinions.^27,28^ Ultimately, the observed differences based on income may lead to inequitable or less patient-centered care, which has the potential to perpetuate health disparities.

Patients who spoke no English at home and patients who preferred their medical care in a language other than English were significantly associated with discordant care in univariate analysis. Similarly, on sensitivity analysis, patients who had insurance other than private insurance were more likely to receive discordant care. However, on multivariate analysis, language and insurance were not found to be a significant predictor of concordance suggesting potentially that income confounded the effect of language and insurance on concordance. While patient income and insurance are not modifiable, language barriers are potentially modifiable from the organizational level, as there are changes an organization can make to mitigate or accommodate for these barriers. Language barriers in particular have been found to strain the patient-surgeon relationship due to difficulty establishing rapport, frustration due to the need for repetition, and the potential for decreased compliance and misinterpretation of treatment plans or instructions.^29,30^ By contrast, patient-physician language concordance is associated with increased patient satisfaction and understanding.^31^ Although the present study was not powered for language and insurance specifically, the results highlight the need for further investigation exploring the impact of language and insurance on goal concordant care which additionally has implications for the patient-surgeon relationship and health equity.

The results should be viewed in light of the limitations. We recognize that multiple definitions of goal concordant care exist and have been studied. While the results may have varied if goal concordance was defined as patient goal matching physician goal or patient goal matching physician-defined treatment, we aimed to understand goal concordant care as defined by the patient. While not formally validated, the treatment list was piloted with patients for comprehension and completeness. Furthermore, the mean satisfaction score was 97% and the average LTR score was 4.8/5, introducing a potential ceiling effect. To account for this, we identified the 33rd percentile as a cutoff for satisfaction based on previously published methodology.^9,19^ In addition, the Observer OPTION5 tool uses subjective measurement. However, our team established interrater reliability and addressed inconsistencies in scoring. The patient population for the present study was recruited from 2 academic institutions in one geographic region and included 58% white participants. As a result, application of results may be limited among other geographic regions and/or among more diverse patient populations. In addition, we recognize there may be factors other than goal concordance that may impact a patient’s satisfaction or experience, and factors we did not include may be associated with goal concordant care. Last, we recognize that patient satisfaction and experience in orthopedic hand surgery were evaluated within a single office visit. Patient satisfaction and experience may be associated with goal concordant care when evaluated longitudinally after multiple clinic visits and patient-surgeon interactions.

Despite these limitations, we demonstrate that while lower income patients had a greater odds of receiving discordant care, concordance was not associated with patient satisfaction or experience. This work highlights the need for optimized delivery of equitable care in these patient populations and the importance of understanding patients’ pretreatment goals and may help inform interventions to address care inequities. Further investigation is warranted to better understand the drivers of goal concordant care and to characterize the impact of the observed associations of wealth disparities on goal concordant care.
